# Deciphering molecular bridges: Unveiling the interplay between metabolic syndrome and Alzheimer’s disease through a systems biology approach and drug repurposing

**DOI:** 10.1371/journal.pone.0304410

**Published:** 2024-05-29

**Authors:** Zahra Azizan, Hakimeh Zali, Seyed Amir Mirmotalebisohi, Maryam Bazrgar, Abolhassan Ahmadiani

**Affiliations:** 1 School of Medicine, Shahid Beheshti University of Medical Sciences, Tehran, Iran; 2 Department of Tissue Engineering and Applied Cell Sciences, School of Advanced Technologies in Medicine, Shahid Beheshti University of Medical Sciences, Tehran, Iran; 3 Student Research Committee, School of Advanced Technologies in Medicine, Shahid Beheshti University of Medical Sciences, Tehran, Iran; 4 Cellular and Molecular Biology Research Center, Shahid Beheshti University of Medical Sciences, Tehran, Iran; 5 Neuroscience Research Center, Shahid Beheshti University of Medical Sciences, Tehran, Iran; Concordia University, CANADA

## Abstract

The association between Alzheimer’s disease and metabolic disorders as significant risk factors is widely acknowledged. However, the intricate molecular mechanism intertwining these conditions remains elusive. To address this knowledge gap, we conducted a thorough investigation using a bioinformatics method to illuminate the molecular connections and pathways that provide novel perspectives on these disorders’ pathological and clinical features. Microarray datasets (GSE5281, GSE122063) from the Gene Expression Omnibus (GEO) database facilitated the way to identify genes with differential expression in Alzheimer’s disease (141 genes). Leveraging CoreMine, CTD, and Gene Card databases, we extracted genes associated with metabolic conditions, including hypertension, non-alcoholic fatty liver disease, and diabetes. Subsequent analysis uncovered overlapping genes implicated in metabolic conditions and Alzheimer’s disease, revealing shared molecular links. We utilized String and HIPPIE databases to visualize these shared genes’ protein-protein interactions (PPI) and constructed a PPI network using Cytoscape and MCODE plugin. SPP1, CD44, IGF1, and FLT1 were identified as crucial molecules in the main cluster of Alzheimer’s disease and metabolic syndrome. Enrichment analysis by the DAVID dataset was employed and highlighted the SPP1 as a novel target, with its receptor CD44 playing a significant role in the inflammatory cascade and disruption of insulin signaling, contributing to the neurodegenerative aspects of Alzheimer’s disease. ECM-receptor interactions, focal adhesion, and the PI3K/Akt pathways may all mediate these effects. Additionally, we investigated potential medications by repurposing the molecular links using the DGIdb database, revealing Tacrolimus and Calcitonin as promising candidates, particularly since they possess binding sites on the SPP1 molecule. In conclusion, our study unveils crucial molecular bridges between metabolic syndrome and AD, providing insights into their pathophysiology for therapeutic interventions.

## Introduction

Alzheimer’s disease (AD) is a progressive neurodegenerative disorder defined by β-amyloid plaques and neurofibrillary tangles accumulation. AD progresses from the hippocampus through the frontal and other brain regions, leading to gradual cognitive impairment and memory loss [1]. It is the most common cause of dementia, and its impact on society is significant, with an estimated 66 million affected in 2030 and double every 20 years [2]. Growing research has linked metabolic syndrome to the progression of AD, making it a significant risk factor to address[1].

Metabolic syndrome (MetS) is a cluster of conditions that occur together, including high blood pressure, high blood sugar, excess body fat around the waist, and abnormal cholesterol or triglyceride levels. These conditions increase the risk of developing cardiovascular disease, stroke, non-alcoholic fatty liver disease (NAFLD), and diabetes (DM) [3].

Studies have found that metabolic syndrome can also contribute to the progression of AD. For example, insulin resistance, a hallmark of metabolic syndrome, has been linked to the accumulation of beta-amyloid protein in the brain, a key feature of AD [4]. Insulin resistance can also lead to inflammation in the brain, which can further contribute to the progression of AD [4]. Epidemiological studies further support the data by reporting a 2-fold increased risk of AD in DM [5, 6]. Additionally, high blood pressure and abnormal cholesterol levels have been associated with cognitive decline and an increased risk of AD [5, 6].

Two classes of medications have been approved for AD to relieve the symptoms: NMDA receptor antagonists (memantine) and cholinesterase enzyme inhibitors (rivastigmine, galantamine, and donepezil) [7]. Developing a cure for Alzheimer’s disease has been challenging due to the complex nature of the disease. However, recent research has focused on targeting MetS as a modifiable risk factor for AD. For example, drugs that target insulin resistance have shown promise in reducing the accumulation of beta-amyloid protein in the brain [8]. Additionally, drugs that target high blood pressure and abnormal cholesterol levels may also be effective in reducing the risk of AD [9, 10]. Further research is needed to fully understand the pathways linking metabolic syndrome and AD. However, targeting metabolic syndrome as a modifiable risk factor may hold promise in developing new treatments and drugs for AD [6].

Identifying the molecular links between AD and MetS may provide insight into the shared molecular pathways involved in the development and progression of both conditions [11].

In recent decades, systems biology approaches, such as protein-protein interaction (PPI) and gene regulatory network (GRN) analyses, have revolutionized the understanding of molecular mechanisms underlying biological phenomena and pathologies [12]. These innovative approaches have been instrumental in unraveling the intricate molecular connections and relationships among various diseases [13].

In this study, we have used a systems biology approach to design a protein-protein interaction network of AD and diseases related to Mets. Besides, this approach allowed us to identify the essential genes and molecular pathways underlying the link between Alzheimer’s and metabolic syndrome and shed light on potential therapeutic targets for these diseases.

## Method

### Study design

**Fig 1:**
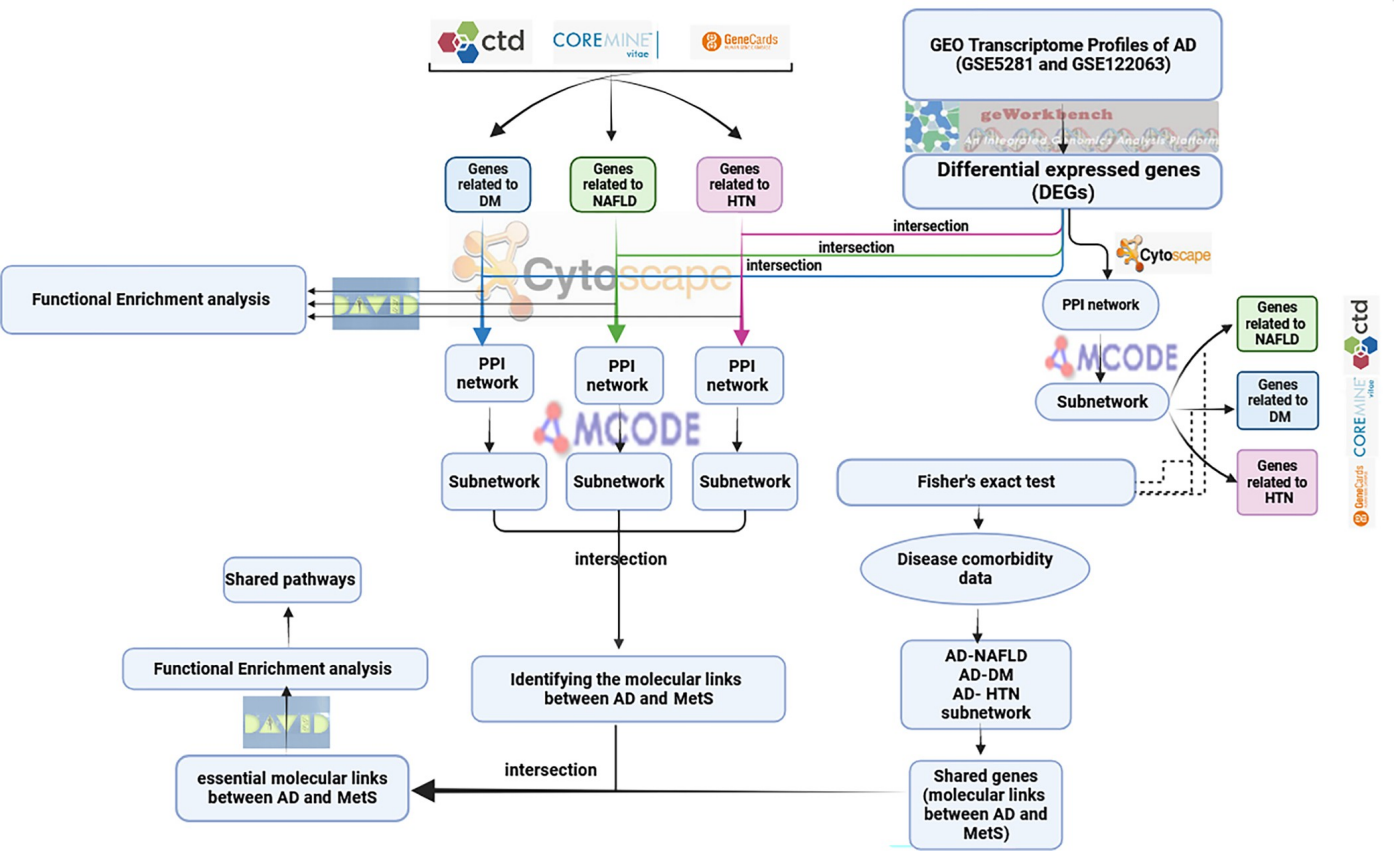
Workflow algorithm. Workflow for constructing a protein-protein interaction network. DM, diabetes mellitus; HTN, hypertension; NAFLD, non-alcoholic fatty liver disease; AD, Alzheimer’s disease; GEO, Gene Expression Omnibus; DE, differentially expressed; GO, Gene Ontology; KEGG, Kyoto Encyclopedia of Genes and Genomes; PPI, protein‑protein interaction network.

We used bioinformatics approaches to identify dysregulated genes in AD and MetS, including DM, hypertension, and NAFLD.Then, we intersected Alzheimer’s DEG and diabetes, hypertension, and NAFLD as metabolic conditions separately. Then, three protein-protein interaction (PPI) networks of shared genes between both AD and MetS conditions were constructed.Ultimately, we analyzed the data to identify the essential molecular links between AD and MetS by finding the shared genes through PPI networks. To validate the results, A PPI network of differentially expressed genes (DEGs) in AD was constructed. MCODE Plugin was used to identify highly connected regions, and the associations between AD clusters and MetS-related genes were evaluated using Fisher’s exact test. Finally, we enriched the shared genes between both conditions (AD and Mets) and the molecular links. Additionally, drug repurposing was applied for the molecular links. An outline of the workflow of this study is summarized in **Fig 1**.

### Data collection

#### GEO data collection and analysis of differentially expressed Genes (DEGs)

We searched the GEO (https://www.ncbi.nlm.nih.gov/geo/) for microarray datasets AD using the following search criteria: "Alzheimer’s disease" and "microarray". We then applied inclusion criteria to select GSE5281 and GSE122063, which met the following criteria: (1) homo sapiens, (2) brain tissue, (3) AD patients and healthy controls≥60 yrs., and (4) microarray platform.

GSE5281 contains 87 patients and 74 control samples and is based on the GPL570 ([HG-U133_Plus_2] Affymetrix Human Genome U133 Plus 2.0 Array) platform. GSE122063 contains 56 Alzheimer’s disease patient and 44 control samples and is based on the GPL16699 (Agilent-039494 SurePrint G3 Human GE v2 8x60K Microarray 039381) platform.

In the present study, we used Ge-Workbench to normalize expression data and filter DEGs between AD and healthy control samples. The DEGs were screened according to the cut-off criteria of adjusted p-value < 0.05 and |log2 (fold change) | >1. Then, we visualized normalized expression data and DEGs of both GSEs with a boxplot and volcano plot using R software. The probe IDs were converted to gene symbols using the GPLs. Then, intersected DEGs between GSE5281 and GSE122063 were identified with the Venn diagram tool(https://bioinformatics.psb.ugent.be/webtools/Venn/). A heatmap was constructed to visualize cluster analysis results of the DEGs.

#### Text data mining

The genes related to DM, hypertension, and NAFLD were obtained using the intersection of three experimental databases: Gene Cards [https://www.genecards.org/], CTD [https://ctdbase.org/], and Core Mine [https://coremine.com/medical/#search]. We used the search criteria "DM" OR "diabetes mellitus", "HTN" OR "hypertension", "NAFLD" OR "non-alcoholic fatty liver disease" to find genes associated with DM, HTN, and NAFLD. Then, the Venn diagram tool extracted the intersected genes of DM, HTN, and NAFLD.

### PPI Network construction and identification of MCODE clusters

#### PPI network of shared genes between AD and MetS

To explore the molecular links between AD and MetS, the PPI network of shared genes between AD and each of the MetS conditions (DM, Hypertension, NAFLD) was separately constructed using the online databases STRING (https://string-db.org/) and Human Integrated Protein-Protein Interaction reference (HIPPIE; http://cbdm-01.zdv.uni-mainz.de/~mschaefer/hippie/) with a cut-off value of a confidence score >0.4. To analyze the data and identify the clusters, the networks from both platforms were imported into Cytoscape (version 3.10) and merged. Any isolated nodes in the merged networks without connections were removed. The Molecular Complex Detection (MCODE) plugin in Cytoscape was used to identify the clusters (Degree cut-off ≥5, node score cut-off ≥2, K‑core ≥2, and max depth = 100) of three PPI networks. Finally, we determined the intersection of genes within the highest-scoring clusters across the three PPI networks to identify potential molecular links between AD and metabolic syndromes.

#### PPI network of AD-related genes and identifying the relationship of its clusters with MetS

To validate the obtained outcomes, a PPI network of differentially expressed genes (DEGs) in AD was constructed, an MCODE Plugin was used to identify highly connected regions, and the associations between AD clusters and MetS-related genes were evaluated using the Fisher’s exact test (https://www.socscistatistics.com/tests/fisher/default2.aspx) with the P-value<0.05 as the significant. This validation strengthens our results and enables us to identify genes that exhibit strong connections and are shared between Alzheimer’s disease and metabolic syndrome.

#### Functional enrichment analysis

The molecular links and the overlapping genes between AD and metabolic-related conditions (DM, Hypertension, and NAFLD) were subjected to gene ontology (GO) and Kyoto Encyclopedia of Genes and Genomes (KEGG) pathway enrichment analyses using the Database for Annotation, Visualization, and Integrated Discovery (DAVID: https://david.ncifcrf.gov/). The GO items consist of 3 parts: biological process (BP), molecular functions (MF), and cellular components (CC). P-value <0.05 was considered statistically significant.

#### Potential therapeutic drugs for essential molecular links between AD and MetS

Based on the essential molecular links between AD and MetS, The DGIdb (https://www.dgidb.org/) was utilized to predict candidate therapeutic drugs for AD.

## Result

### Data pre-processing and identification of AD-associated DEGs

**Fig 2:**
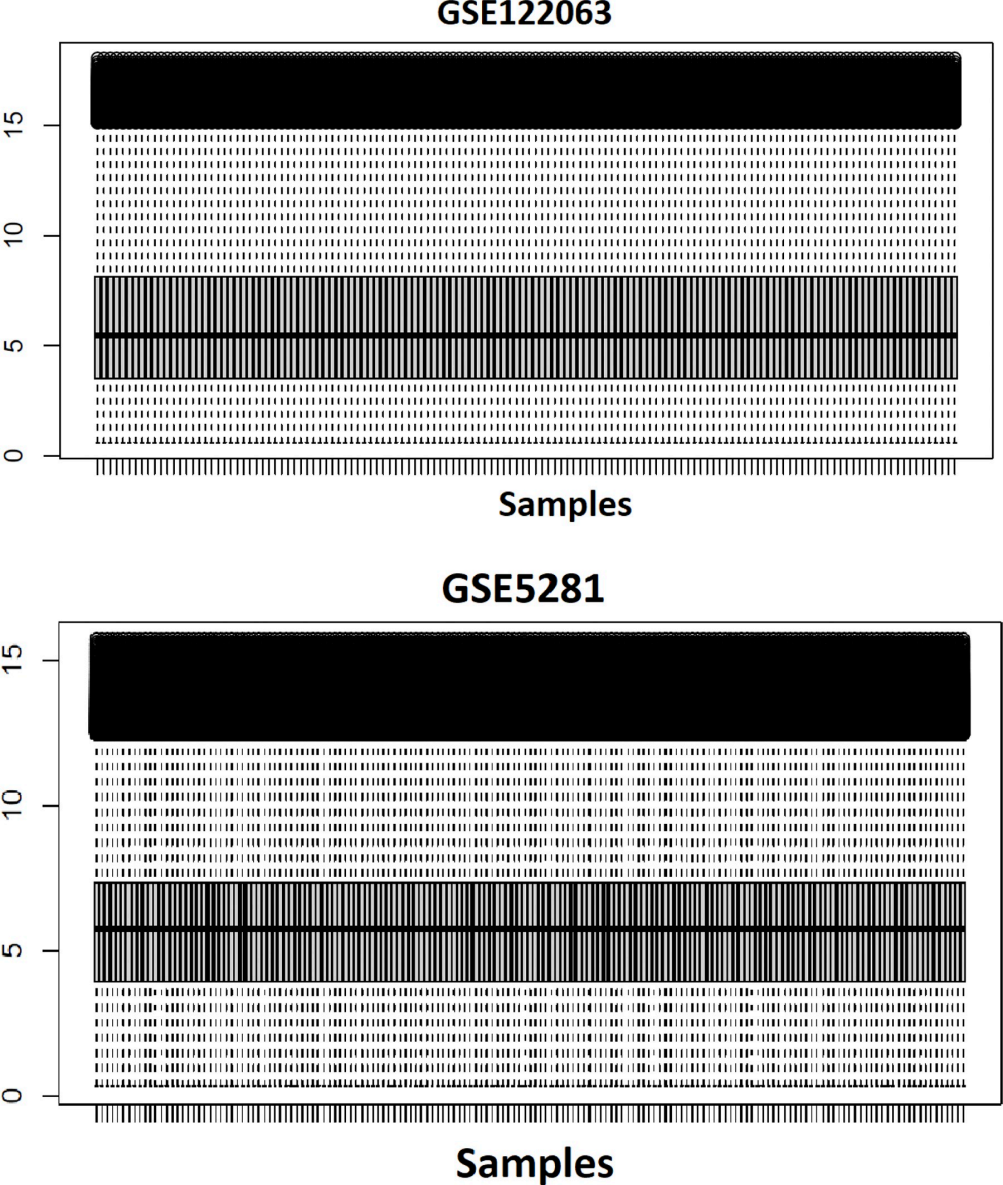
Identification of DEG. The boxplots show the normalized data from GSE122063 (A) and GSE5281 (B). The volcano plots of GSE122063 (C) and GSE5281 (D) highlight the upregulated and downregulated genes in the Alzheimer’s group vs the healthy group, represented by red and blue colors, respectively. GSE122063 (E) and GSE5281 (F) heatmaps reveal gene expression patterns. Using the Venn diagram, 141 common differentially expressed genes (DEGs) were identified between the two datasets (G), indicative of Alzheimer’s disease.

The datasets from GSE 5281 and GSE122063 were subjected to normalization using Ge-workbench. Two boxplots of normalized data from both GSEs are shown in **Fig 2A and 2B**, respectively. These boxplots show that the expression data quality was reliable and the normalization was sound. Genes with a log fold change (Ⅰlog FCⅠ) greater than one and an adjusted p-value < 0.05 were selected as differentially expressed genes (DEGs). In GSE5281, 2424 DEGs were identified, comprising 1514 upregulated genes and 910 downregulated genes. In GSE122063, 904 genes were identified as DEGs, among which 311 were upregulated, and 593 were downregulated. The expression of all DEGs from both GSEs is shown in two volcano plots (**Fig 2C and 2D**). Using the Venn diagram tool, 141 common genes were identified as AD-associated DEGs, consisting of 43 upregulated and 98 downregulated genes (**Fig 2G, S1 File**). Two heatmaps visualize the cluster analysis results of the DEGs of both GSE (**Fig 2E and 2F**).

### Identification of genes associated with MetS conditions and shared with AD

**Fig 3:**
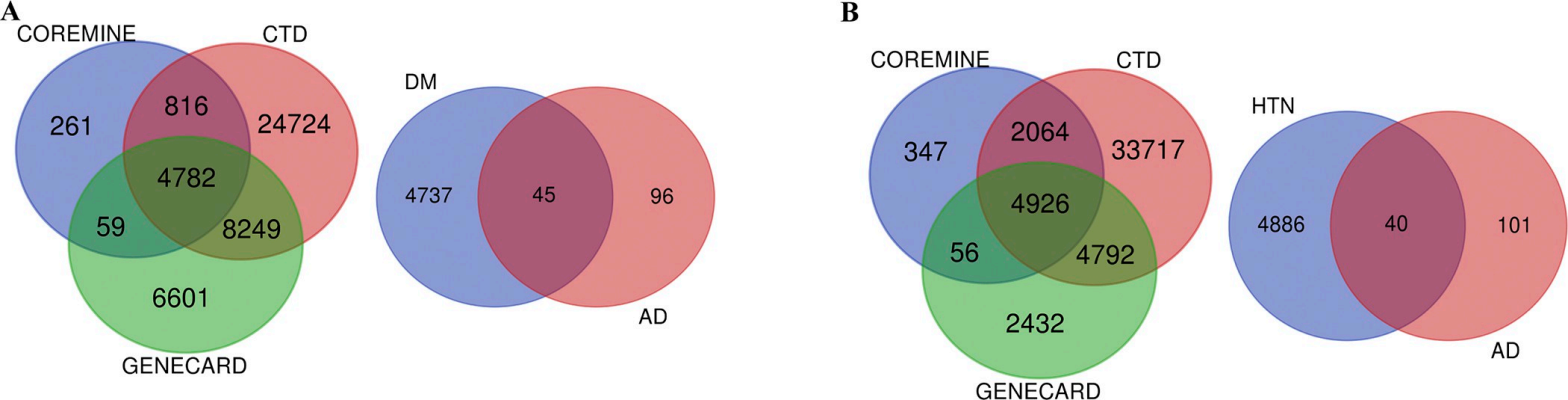
Intersected genes. Intersection of shared genes between DM(A), HTN(B), and NAFLD(C) from the text-mine datasets (Core Mine, CTD, Gene Cards) with Alzheimer’s disease-related DEGs using the Venn diagram tool. DM, diabetes mellitus; HTN, hypertension; NAFLD, non-alcoholic fatty liver disease; AD, Alzheimer’s disease.

We conducted text mining on three datasets, Core mine, Gene cards, and CTd, to extract gene information from MetS-related diseases. In the case of DM, we extracted a total of 19,691 genes from Gene cards, 277,451 genes from CTd, and 5,918 genes from Core mine. Using the Venn diagram tool, we identified an intersection of 4,782 genes specific to DM. Similarly, for hypertension, we extracted 12,206 genes from Gene cards, 157,023 genes from CTd, and 7,393 genes from Core mine, and found an intersection of 4,926 genes relevant to hypertension. Concerning non-alcoholic fatty liver disease (NAFLD), we extracted 3,129 genes from Gene cards, 36,789 genes from CTd, and 4,031 genes from Core mine. Among these, 1,148 genes were found to be intersected and associated with NAFLD. Furthermore, we intersected the AD-associated DEGs, which consisted of 141 genes, with the genes associated with MetS conditions. Out of these, 45 genes were found to be common between DM and AD, 40 genes were common between hypertension and AD, and 10 genes were common between NAFLD and AD (**Fig 3, S2 File**).

### PPI network of shared genes between AD and MetS conditions and identification of molecular links

**Fig 4:**
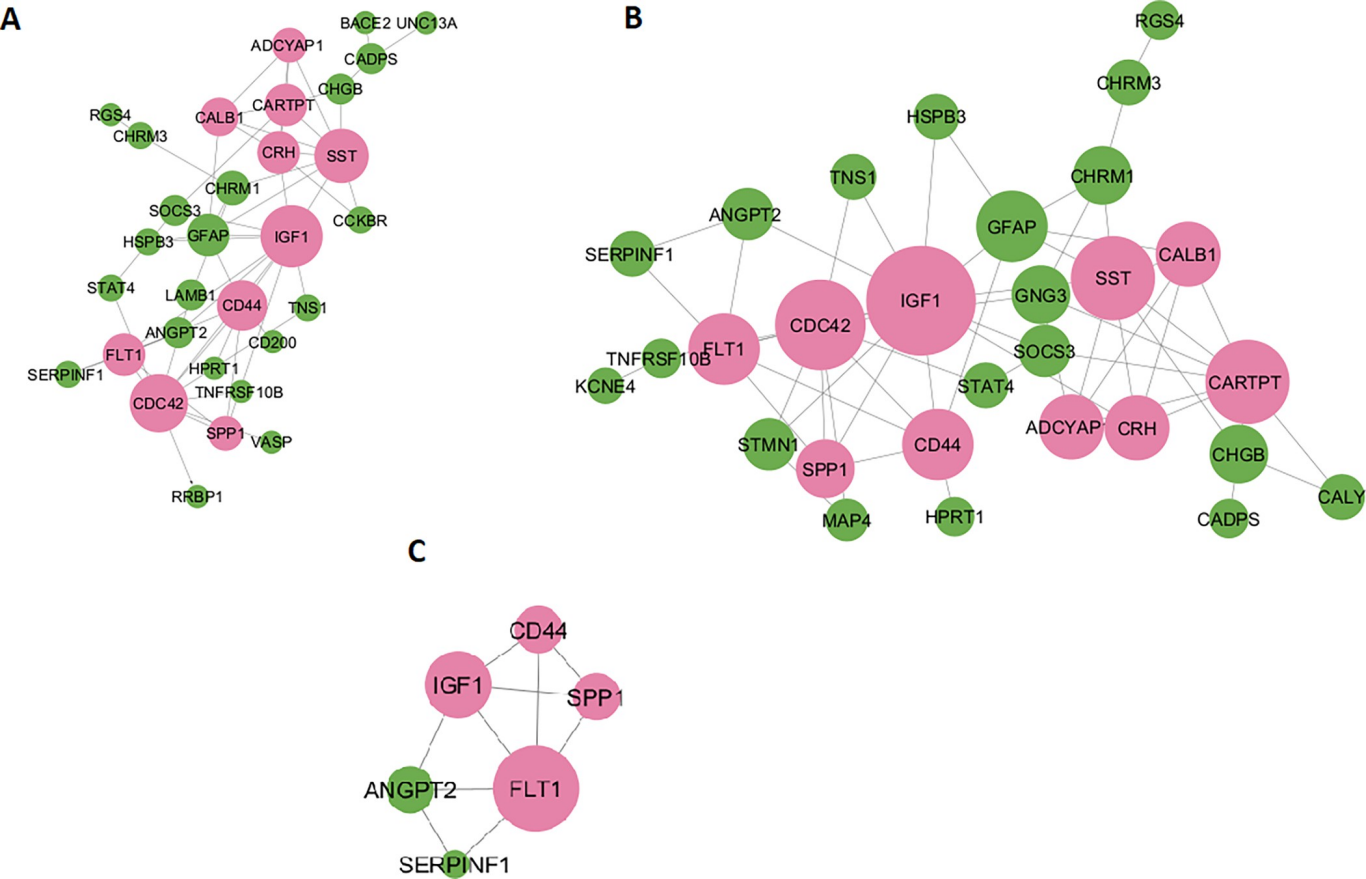
Protein-protein interaction network of metabolic syndrome conditions and Alzheimer. The protein-protein interaction networks of AD and MetS-related diseases include DM(A), HTN (B), and NAFLD (C). The size of the nodes represents their degree of connectivity within the network. Pink circles highlight clusters of interconnected nodes, while the green ones symbolize nodes outside the clusters.

The PPI networks of 40 shared genes between AD and hypertension displayed 29 nodes and 58 edges, consisting of a cluster of 10 highly connected genes (**Fig 4A**). Similarly, the PPI networks of 45 shared genes between AD and diabetes mellitus (DM) exhibited 31 nodes and 87 edges, including ten genes forming a cluster (**Fig 4B**). Additionally, we discovered that non-alcoholic fatty liver disease (NAFLD) and AD were connected by six nodes and ten edges, along with a cluster formed by four genes (**Fig 4C**).

Consequently, we have identified FLT1, SPP1, CD44, and IGF1 as the critical genes shared among the clusters in the PPI network of AD and metabolic syndrome. These molecular links exhibit significant network connectivity and display altered expression levels concerning Alzheimer’s disease. (**Fig 4A–4C**).

### Identifying clusters in the AD PPI network and associating them with MetS conditions-related genes

**Fig 5:**
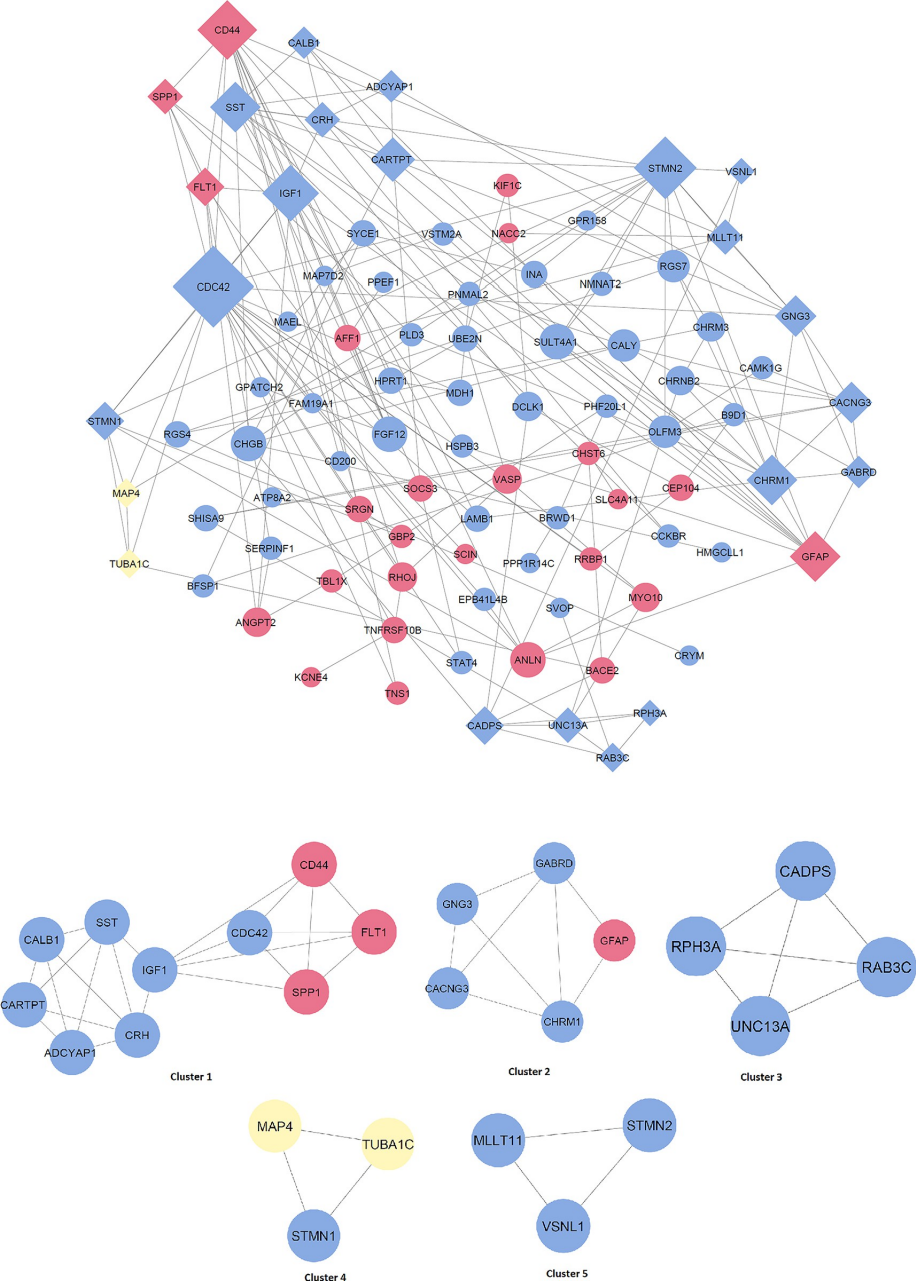
PPI network of Alzheimer. The Alzheimer’s PPI network comprises 89 nodes and 182 edges. In this network, upregulated genes are denoted by red circles, while blue circles represent downregulated genes. The yellow nodes indicate genes exhibiting distinctive regulation patterns between the GSE5281 and GSE122063 datasets. The network shows five distinct clusters constructed by merging the HIPPIE and String databases. The size of the nodes corresponds to their level of connectivity. A group of diamond shapes depicts nodes that are connected and form a cluster.

Based on our analysis of the protein-protein interaction (PPI) networks of the 141 differentially expressed genes (DEGs) in Alzheimer’s disease (AD), we have identified 5 clusters using the MCODE algorithm, as shown in **Fig 5**. To validate the crucial genes that establish a connection between metabolic syndrome and AD, we performed the Fisher exact test.

The first cluster of genes comprises ten genes associated with diabetes mellitus (DM) and hypertension. Still, only four of these genes are connected to non-alcoholic fatty liver disease (NAFLD). The Fisher exact test yielded a significant result for the first cluster, suggesting that these genes may be crucial in linking metabolic syndrome to Alzheimer’s disease (**Table 1**). However, no significant associations were observed for the remaining clusters.

**Table 1:** The Fisher’s exact test.

**Genes**	**Shared genes (AD+ DM)**	**Non-Shared genes (AD- DM)**	**Total number**
Genes inside the cluster1	10	0	10
Genes outside the cluster1	35	96	131
Total genes	45	96	**141 = total DEGs of AD**
**Genes**	**Shared genes (AD+ HTN)**	**Non-Shared genes (AD- HTN)**	**Total number**
Genes inside the cluster1	10	0	10
Genes outside the cluster1	30	101	131
Total genes	40	96	**141 = total DEGs of AD**
**Genes**	**Shared genes (AD+ NAFLD)**	**Non-Shared genes (AD- NAFLD)**	**Total number**
Genes inside the cluster1	4	6	10
Genes outside the cluster1	6	125	131
Total genes	10	131	**141 = total DEGs of AD**

The p-values for all groups were found to be <0.00001, while the cut-off p-value was set at <0.05. AD, Alzheimer’s disease; DM, Diabetes Mellitus; HTN, hypertension; NAFLD, non-alcoholic fatty liver disease; DEG, differentially expressed genes.

### Functional enrichment analysis of shared genes between AD and MetS conditions

**Fig 6:**
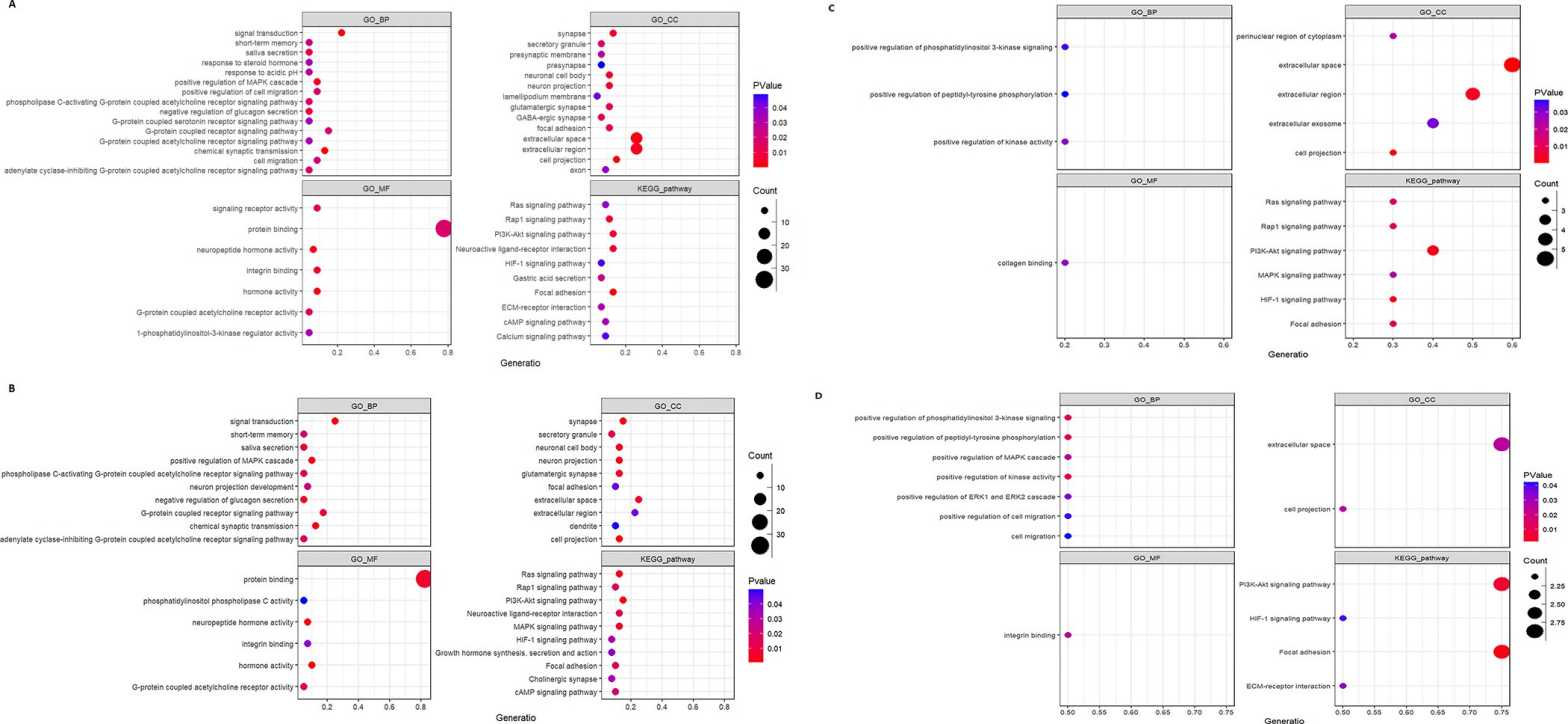
Functional enrichment analysis. Functional enrichment analysis was conducted to analyze the shared genes between Alzheimer’s disease (AD) and metabolic diseases, namely diabetes (A), hypertension (B), and non-alcoholic fatty liver disease (NAFLD) (C). Additionally, the enrichment analysis of molecular links between AD and MetS (D) was demonstrated.

The results of our analysis are summarized in **Fig 6.** The **functional enrichment** analysis of shared genes between AD and diabetes mellitus (DM) reveals that the significantly enriched BP terms were mainly associated with chemical synaptic transduction and signal transduction, which are implicated in synaptic dysfunction. Furthermore, the enrichment includes processes such as positive regulation of the MAPK cascade involved in synaptic plasticity, learning, and memory formation and the negative regulation of glucagon and saliva secretion (**Fig 6A**).

Regarding cellular component (CC), the significant enrichment involves cell projections, the extracellular space and region, synapses, and neuronal cell bodies (**Fig 6A**). Moving on to molecular function (MF), the enriched items are mainly associated with hormone activity, neuropeptide hormone activity, integrin binding, signaling receptor activity, and G-protein coupled acetylcholine receptor activity (**Fig 6A**).

Furthermore, the KEGG pathway analyses indicate that the shared genes between AD and DM are primarily involved in various pathways. These pathways include the focal adhesion pathway, which connects the extracellular matrix to the cell’s cytoskeleton. Disruptions in this pathway can affect neuron stability and function, impairing synaptic function and neuronal degeneration. The rap1 signaling pathway, which regulates cellular processes like cell adhesion, proliferation, and differentiation, is also involved. The PI3K-Akt signaling pathway also plays a crucial role in cell survival, growth, and metabolism. It is implicated in multiple cellular processes, including neuronal survival, synaptic plasticity, and regulation of protein synthesis. The other enriched pathways include neuroactive ligand-receptor interaction and gastric acid secretion (**Fig 6A**).

Similar results were observed in the shared genes between hypertension and AD enrichment analysis compared to the DM-AD network analysis in terms of GO analysis and KEGG pathways. Additionally, other notable pathways included the Ras signaling pathway and MAPK signaling pathway (**Fig 6B**).

We also analyzed NAFLD and AD, which yielded noteworthy findings. The enrichment analysis revealed several significant biological processes, including the positive regulation of kinase activity, positive regulation of phosphatidylinositol 3-kinase signaling (PI3K signaling), and positive regulation of peptidyl-tyrosine phosphorylation. In Alzheimer’s disease, PI3K signaling can impact multiple aspects of disease pathology, such as beta-amyloid production, synaptic function, neuronal survival, and tau pathology. Also, dysregulation of peptidyl-tyrosine phosphorylation affects various cellular processes, including neuronal survival, synaptic plasticity, and memory formation (**Fig 6C**).

Furthermore, the CC analysis identified shared genes involved in the extracellular space, cell projection, extracellular region, perinuclear region of the cytoplasm, and extracellular exosome (**Fig 6C**). Regarding molecular function (MF) analysis, only two shared genes were identified: AEBP1 and CD44, which are involved in collagen binding (**Fig 6C**).

Our analysis also revealed significant KEGG pathways, including the PI3K-Akt signaling pathway, the HIF-1 signaling pathway (which may contribute to neuroinflammation, oxidative stress, and neuronal damage), focal adhesion, and the Ras signaling pathway. (**Fig 6C**).

### Functional enrichment analysis of essential molecular links between AD and MetS

Furthermore, the enrichment analysis of key genes, which serve as the molecular links between Alzheimer’s and metabolic syndrome, revealed the focal adhesion pathway, PI3K-Akt signaling pathway, ECM-receptor interaction (ECM-receptor interactions can impair cell signaling pathways, promote neuroinflammation, and contribute to the progression of AD pathology), and the HIF-1 signaling pathway as the notable pathways. The significant biological processes included positive regulation of kinase activity, positive regulation of phosphatidylinositol 3-kinase signaling, and positive regulation of peptidyl-tyrosine phosphorylation. For the cellular component, the significant findings included extracellular space and cell projection, while integrin binding was suggested in the MF analysis of molecular linked genes (**Fig 6D**).

### Potential therapeutic drugs for essential molecular links between AD and MetS

**Fig 7:**
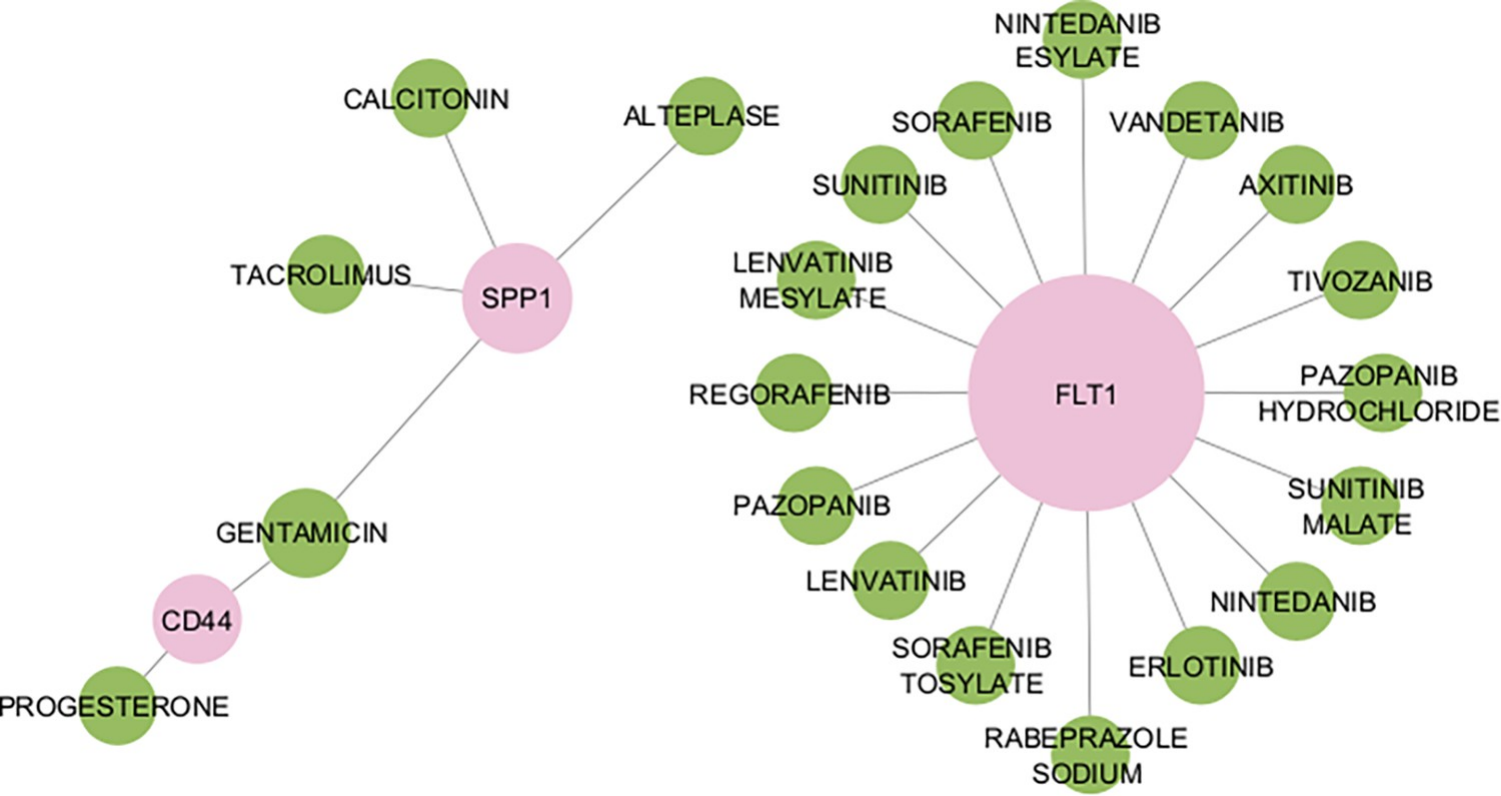
Drug repurposing of molecular links. Repurposing Essential Molecular Linked Genes for Drug Discovery. Visualized by pink circles representing the genes and green ones symbolizing approved targeted drugs.

We utilized the drug-gene interaction database (DGIdb) to identify potential drugs for treating Alzheimer’s disease based on the pathogenesis and drug-gene interaction. Based on the database, we discovered 22 approved drugs for the gene FLT1, four for SPP1, and two for CD44. However, we found no drug interactions specifically related to the gene IGF1(**Fig 7**).

## Discussion

Metabolic syndrome has emerged as a significant risk factor for Alzheimer’s disease, sharing common pathological features such as mitochondrial dysfunction, insulin resistance, and oxidative stress [4, 14]. Although previous studies have shed light on the relationship between Alzheimer’s and metabolic syndrome, our understanding of the molecular mechanisms that connect these diseases remains incomplete. In our study, we aimed to unravel the intricate molecular links between Alzheimer’s and metabolic syndrome to advance our knowledge of the pathogenesis of these conditions. Our analysis identified four essential genes—SPP1, IGF1, CD44, and FLT1—that serve as potential molecular links between Alzheimer’s and metabolic syndrome.

Studies have shown that extracellular SPP1 acts as a chemotactic factor and cytokine to activate microglia and initiate the neuroinflammatory process. Specifically, SPP1 secreted by perivascular macrophages induces microglia phagocytosis through various pathways, including opsonization of neurons and activation of macrophages and microglia for synaptic engulfment [15, 16]. While the exact mechanism of SPP1 in Alzheimer’s pathogenesis is not yet fully understood, it is believed that SPP1 plays a significant role in neuroinflammation [17].

SPP1 can also be traced in metabolic syndrome (MetS) through its role in conditions such as diabetes, non-alcoholic fatty liver disease (NAFLD), and hypertension [18–20]. For instance, research has shown that elevated blood glucose levels increase SPP1 secretion, which can contribute to insulin resistance by promoting chronic low-grade inflammation and interfering with insulin signaling pathways like PI3K/Akt, which plays a key role in MetS progression, especially diabetes. Additionally, elevated SPP1 levels contribute to the progression of diabetes through beta-cell apoptosis [16, 19, 21].

SPP1 has also been upregulated in the liver tissues of patients with NAFLD. It is believed to be involved in inflammation, fibrosis, and regulation of lipid metabolism in the liver [20, 22]. Obesity and high blood glucose through elevated SPP1 can induce insulin resistance, atherosclerosis, and diabetes [18].

The role of elevated SPP1 in vascular inflammation and endothelial dysfunction provides insight into how blood-brain barrier dysfunction may contribute to neuroinflammation and the development of Alzheimer’s disease (AD). Elevated SPP1 levels may also be linked to vascular stiffness and hypertension [16, 18, 23]. Our functional enrichment analysis has revealed that SPP1 may interact with integrin and CD44 in the extracellular matrix, leading to inflammation through the activation of the PI3K/Akt pathway. This pathway is crucial in inflammation and is consistent with recent studies [16, 24].

Consistent with our results, findings have suggested that IGF1, FLT1, and CD44 may be involved in the development and progression of Alzheimer’s disease and MetS. IGF1 is crucial in neuronal development, synaptic plasticity, and neuroprotection. It has been implicated in regulating glucose metabolism and insulin signaling [25]. Lower levels of IGF1 have been associated with an increased risk of developing Alzheimer’s disease [26]. On the other hand, CD44 is a cell surface protein involved in various cellular processes, including cell adhesion and migration. Studies have suggested that CD44 may be involved in the development and progression of metabolic syndrome. CD44 has been shown to promote inflammation and insulin resistance, which are vital components of metabolic syndrome [27].

Furthermore, CD44 has been implicated in the pathogenesis of Alzheimer’s disease through its involvement in immune system regulation [28]. Also, FLT1, known as vascular endothelial growth factor receptor 1, is involved in angiogenesis. It has also been implicated in insulin resistance [29] and neurodegenerative processes [30]. These findings highlight the possible interplay between cellular processes in the development of both Alzheimer’s disease and metabolic syndrome [31].

According to our functional enrichment analysis, these four signaling pathways, namely PI3k/Akt, HIF-1, ECM receptor interaction, and focal adhesion signaling, play a crucial role in the development of Alzheimer’s disease and metabolic syndrome [24, 32–34]. Studies have shown that the ECM, through OPN/SPP1 binding integrins and CD44, can activate focal adhesion signaling [24, 34, 35] and PI3K/Akt pathways [21, 36, 37], which are involved in inflammation, glucose metabolism, and the buildup of amyloid plaques. Lastly, HIF-1 is also activated under conditions of oxidative and inflammatory stress, exacerbating the inflammatory processes associated with Alzheimer’s disease and metabolic syndrome [32, 38, 39].

In summary, the relationship between Alzheimer’s disease and metabolic syndrome is complex and multifaceted. Inflammation appears to play a significant role in the development of both conditions. It is believed that metabolic disorders, such as hyperlipidemia and hyperglycemia, can trigger systemic and local inflammation by producing inflammatory cytokines. This inflammation in the brain activates microglia, leading to synaptic dysfunction and neuronal loss [1].

Furthermore, insulin resistance, which occurs in peripheral tissues due to metabolic disorders, disrupts glucose metabolism in the peripheral environment and the brain. Since glucose is the primary fuel for brain cells, this disruption can result in synaptic destruction and the progression of inflammation in Alzheimer’s disease. Insulin also plays a crucial role in neuronal processes such as learning and memory, and the emergence of central insulin resistance can contribute to impairing neuronal and synaptic function [11].

Moreover, hypertension and other metabolic disorders can cause endothelial damage, affecting the integrity of the blood-brain barrier. This damage can lead to increased blood-brain barrier permeability, making brain cells more susceptible to injury and inflammation [23].

Research has revealed that SPP1 binds to integrins and CD44 and interacts with calcium-binding factors and heparin [40]. These findings are in line with our drug repurposing analysis, suggesting that calcitonin and tacrolimus could be potential drug targets [41, 42]. Tacrolimus is currently undergoing phase 2 clinical trials. While its specific molecular target or pathway has not yet been determined, studies have shown that modulating inflammation may contribute to reducing tau aggregation and the progression of Alzheimer’s disease (AD) [41].

While there is limited research exploring the use of calcitonin as a standalone treatment for AD, current data suggest a potential connection between osteoporosis and the development of AD [43]. Recent studies have brought attention to the activation of the calcitonin receptor through the calcitonin gene-related peptide (CGRP), revealing the potential therapeutic effects of calcitonin in Alzheimer’s disease (AD) and metabolic syndrome (MetS). It is attributed to their shared structure and utilization of the same receptor [42].

Some studies have explored the effect of anti-hypertensive, anti-diabetes, and statin drugs on Alzheimer’s disease and found that they may lead to a relative reduction in SPP1 levels [44–48]. While the drug repurposing process may not confirm their effects, it is hypothesized that these drugs may help reduce the risk factors that contribute to elevated SPP1. These findings provide valuable insights into potential drug targets and pathways for treating Alzheimer’s disease and warrant further investigation.

It’s important to acknowledge the limitations of our study. The lack of an experimental model for advanced research may have restricted our ability to confirm the results and thoroughly explore the mechanisms underlying the association between Alzheimer’s disease and metabolic syndrome. Experimental models can provide valuable insights, so future studies should address this limitation.

Another limitation is the lack of sufficient data on metabolic syndrome (Mets). Text mining for genes related to Mets is a helpful approach, but the absence of available Gene Expression Omnibus (GEO) datasets specifically focused on Mets may have limited the scope of our analysis. Obtaining more comprehensive data on Mets would enhance the robustness of our findings.

Additionally, the lack of clear staging information for diabetes, non-alcoholic fatty liver disease (NAFLD), hypertension, and Alzheimer’s disease may have limited our ability to analyze the relationship between these conditions. The mixture of data from different stages might have introduced confounders and made it challenging to draw definitive conclusions. It would be beneficial to better gather stage-specific data in future studies to understand the progression and interplay of these diseases.

## Conclusion

Finally, we concluded that SPP1, IGF1, CD44, and FLT1 may play a significant role in the pathogenesis of both conditions. Therefore, these genes can be considered molecular links or shared genes between Alzheimer’s disease and metabolic syndrome. Furthermore, we suggest that targeted drug therapies for these molecular links, mainly focusing on SPP1, may be a promising approach for treating both Alzheimer’s disease and metabolic syndrome. These findings open up opportunities for novel treatments to address the shared molecular mechanisms underlying these conditions and potentially help patients with comorbidities. Overall, the results provide new insights into the molecular mechanisms underlying the relationship between Alzheimer’s disease and metabolic syndrome and have the potential to guide the development of more effective therapies for patients with both conditions.

## Supporting information

S1 File Differentially expressed genes of AD. Differentially expressed genes associated with Alzheimer’s disease were extracted from two gene expression datasets, GSE5281 and GSE122062.
(XLSX)

S2 File Text-mining of MetS-related diseases. Explore the genetic landscape of MetS-related diseases, including hypertension, diabetes, and non-alcoholic fatty liver disease (NAFLD), through the text mining method and uncover shared genes with differentially expressed genes in Alzheimer’s disease.
(XLSX)

S1 Graphical abstract (TIF)

## Abbreviation

Osteopontin (OPN)/SPP1: secreted phosphoprotein 1
IGF-1: Insulin-like growth factor 1
FLT1: Fms Related Receptor Tyrosine Kinase 1
AD: Alzheimer’s Disease
MetS: metabolic Syndrome
DM: diabetes mellitus
HTN: hypertension
NAFLD: non-alcoholic fatty liver disease
PPI: protein-protein interaction
DEG: differentially expression gene
GEO: Gene Expression Omnibus
GO: Gene Ontology
KEGG: Kyoto Encyclopedia of Genes and Genomes
