# Supplementary figures and images for: Deciphering molecular bridges: Unveiling the interplay between metabolic syndrome and Alzheimer’s disease through a systems biology approach and drug repurposing
Source: PLoS One. 2024 May 29;19(5):e0304410. doi: 10.1371/journal.pone.0304410 (PMC11135670; doi:10.1371/journal.pone.0304410)

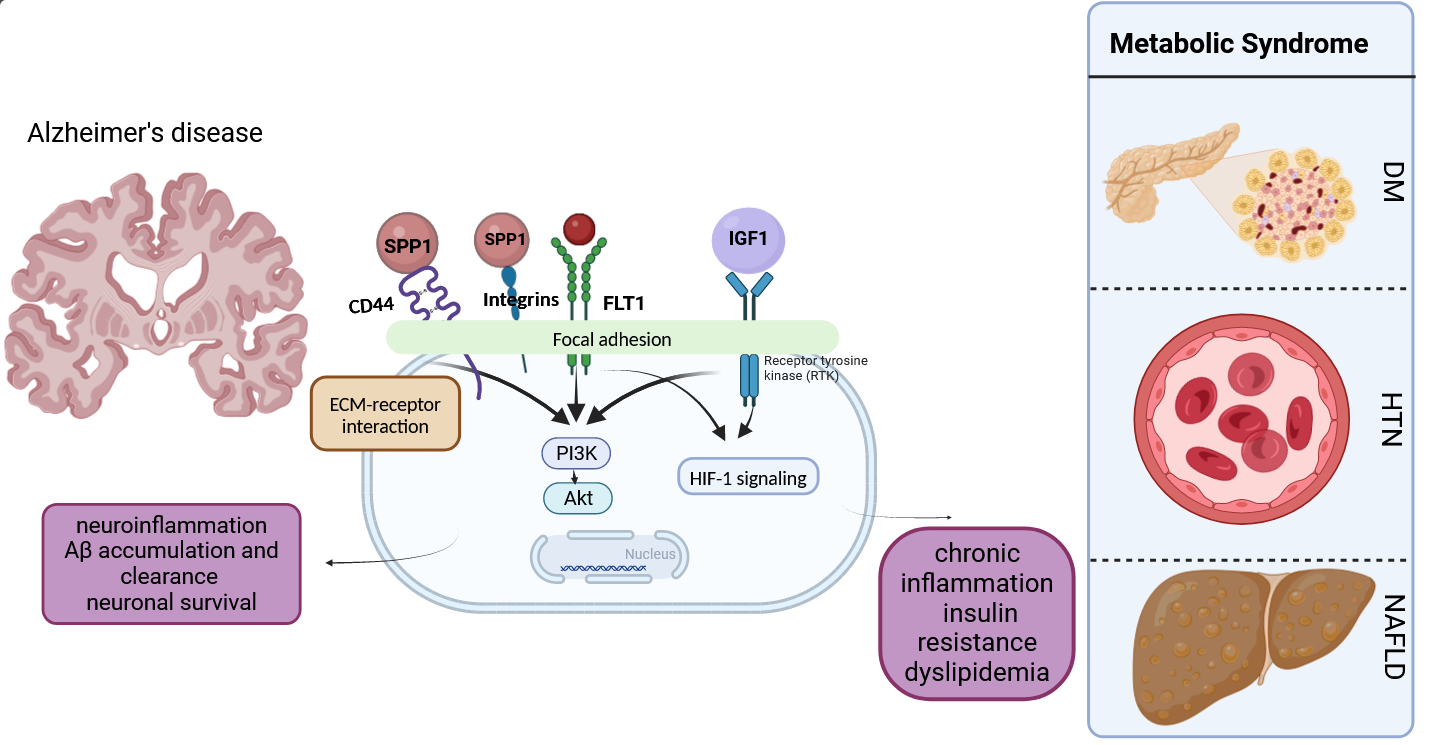

Supplement: S1 Graphical abstract — (TIF) [file pone.0304410.s003.tif]
